# Planetary health education in undergraduate medical education in Germany: results from structured interviews and an online survey within the national PlanetMedEd Project

**DOI:** 10.3389/fmed.2024.1507515

**Published:** 2025-02-11

**Authors:** Fabio Grieco, Sandra Parisi, Anne Simmenroth, Michael Eichinger, Janina Zirkel, Sarah König, Jana Jünger, Eva Geck, Eva-Maria Schwienhorst-Stich

**Affiliations:** ^1^Department of General Practice, University Hospital Würzburg, Würzburg, Germany; ^2^Center for Preventive Medicine and Digital Health, Medical Faculty Mannheim, Heidelberg University, Mannheim, Germany; ^3^Institute of Medical Biostatistics, Epidemiology and Informatics, University Medical Center of the Johannes Gutenberg University Mainz, Mainz, Germany; ^4^Department of Internal Medicine II, University Hospital Würzburg, Würzburg, Germany; ^5^Institute of Medical Teaching and Medical Education Research, University Hospital Würzburg, Würzburg, Germany; ^6^CARES Institute, Heidelberg, Germany; ^7^Faculty of Medicine, Heidelberg University, Heidelberg, Germany

**Keywords:** Planetary Health, Planetary Health Education, One Health, climate change and health, health professions education, medical schools, curriculum development, education for sustainable healthcare

## Abstract

**Background:**

In light of the accumulating evidence, awareness and urgency to act upon the three planetary crises – climate change, biodiversity loss, and pollution – the concept of Planetary Health underscores their profound implications for health and promotes actionable solutions to advance both wellbeing and ecological sustainability. Despite (inter)national calls to integrate Planetary Health into health workers’ curricula, the current status of Planetary Health Education in undergraduate medical education in Germany is unclear. This study therefore aimed (a) to assess the current implementation of Planetary Health in undergraduate medical education in Germany and (b) to explore its characteristics as a foundation to develop evidence-informed recommendations for mainstreaming Planetary Health Education in medical schools in Germany.

**Methods:**

The study comprised structured interviews followed by an online survey, both targeting all 39 medical schools in Germany. In 2021, structured interviews were conducted with students, educators and deanery staff at medical schools. In 2023, educators and deanery staff participated in an online survey based on the findings from the interviews.

**Findings:**

In total, 80% of the 39 medical schools participated in the interviews, while 90% took part in the online survey. Based on integrated findings, 35 medical schools (90%) offered Planetary Health Education, with a median of two educational activities, including both stand-alone courses and lectures integrated into other courses. Despite an overall increase since winter semester 2021/2022, most educational activities were electives and not part of the mandatory curriculum. Innovative educational approaches and learning objectives differed significantly between mandatory and elective formats. In contrast to mandatory educational activities, student involvement was reported for the majority of electives and was significantly associated with transformative learning objectives.

**Interpretation:**

Despite a steady rise in teaching activities, mandatory Planetary Health Education remains insufficiently integrated into undergraduate medical education in Germany. Key criteria defining high-quality Planetary Health Education, such as innovative educational approaches, practical skills, and transformative learning, were primarily reflected in electives, that reach only a minority of students. To adequately equip the future healthcare workforce, the current barriers to successfully integrating Planetary Health into medical education must be systematically addressed and overcome.

## 1 Introduction

Climate change and the transgression of other planetary boundaries, such as biodiversity loss, altered biogeochemical flows, and pollution, are the most important health threats in the twenty-first century (1, 2). Planetary Health (PH) is a solutions-oriented field that focuses on the interconnectedness of climate and ecological crises with societal, political, and economic systems, and their impacts on health and well-being (2).

Due to human activities, safe operating spaces have been crossed for six of the nine planetary boundaries, posing significant risks to life as we know it. Climate change, for instance, leads to an increase in heat-related deaths, particularly among vulnerable populations (3). Rising temperatures have a substantial impact on infectious disease patterns, facilitating the spread of vector-borne diseases. Air pollution, closely linked to the combustion of fossil fuels, not only has detrimental effects on respiratory tract diseases such as asthma and COPD, but also serves as a risk factor for cardiovascular and neuropsychiatric conditions (4).

The healthcare sector contributes to climate change, accounting for 4,4% of global greenhouse gas emissions (5). Combined with a rapidly changing burden of disease, this highlights the urgent need to implement sustainable healthcare solutions, and adapt healthcare systems to provide high-quality services for climate- and pollution-related diseases (5, 6). Healthcare workers, leveraging the trust placed in them, can serve as key agents of transformative action (6–8), both in adaptation and mitigation.

The concept of PH extends beyond the climate crisis to encompass other ecological crises. A holistic understanding of PH emphasizes interdisciplinarity, a deep connection with nature, and the inclusion of restorative, Indigenous, and intergenerational perspectives to broaden perspectives beyond an anthropocentric lens (9, 10). The concept of One Health, which promotes the health of humans, animals, and ecosystems—including marine ecosystems—is closely aligned with PH. In this work, we use PH as an inclusive framework that integrates diverse elements from these related concepts.

The need to train medical students in PH and sustainable healthcare by integrating transformative Planetary Health Education (PHE) – which includes education for sustainable health care (ESH) – into medical curricula is increasingly recognized by a growing number of stakeholders (6, 10–12). Despite this, a global survey from 2020 revealed that only 15% of medical schools had incorporated education on climate change and health into their curricula (10). Evidence on the current implementation of PHE and its characteristics remains sparse, primarily consisting of individual case reports (13, 14) or broad overviews (15–17). A structured approach to assess the implementation of PHE is the Planetary Health Report Card (PHRC) (18), a student-driven initiative aimed at evaluating PH activities in health professions’ education. As of 2024, only eight report cards have been submitted for medical schools in Germany across all three annual data collection rounds, providing an incomplete overview of PHE in undergraduate medical education.

The *Lancet Countdown Policy Brief 2021 for Germany* underscored that PHE often relies on individual efforts rather than being institutionally embedded, and further emphasized the critical need to enhance data on PHE (12). The training gap is further reinforced by research suggesting that medical students are aware of the health effects of – in this case – climate change, yet perceive limited implications for their professional responsibilities (19, 20). Moreover, many students feel they receive insufficient training on these topics during their studies and express interest in expanding the integration of PHE in their education (19, 21).

PHE emphasizes Planetary Health literacy as a central educational goal, extending beyond the mere acquisition of knowledge to include values and transformative competencies (6, 8). Achieving this depth in PHE requires diverse didactic approaches that foster not only intellectual understanding but also ethical engagement and skill-based competencies (6, 8, 10). This alignment of educational approaches with different levels of Miller’s competency framework – categorized as “Knows,” “Knows how,” “Shows,” and “Does” (22) – is essential for developing effective PHE (8). While traditional lectures may suffice for foundational knowledge (“Knows,” “Knows how”), they fall short in training higher-level competencies such as “Shows” and “Does,” which require active student engagement. For these advanced competencies, hands-on and reflective educational approaches are essential, enabling learners to train practical skills, including communication, critical to Planetary Health advocacy and action (6–8, 10).

In Germany, the medical degree program spans six years, consisting of two pre-clinical years, three clinical years, and one year of practical training. The Medical Licensing Regulations (*Ärztliche Approbationsordnung*) specify all mandatory subjects in the standard curriculum (23). Planetary Health, however, is notably absent from this list of required subjects.

The content and objectives of medical curricula are outlined in the German National Competency-based Learning Objectives Catalog in Medicine (*Nationaler Kompetenzbasierter Lernzielkatalog Medizin, NKLM*), first introduced in 2015 and currently being updated from Version 2.0 to 3.0 (24). The NKLM provides a comprehensive framework of learning objectives (LO) for each subject within the medical curriculum, offering guidance to medical schools, though it remains non-binding. Beyond the core curriculum, the current version (NKLM 2.0) includes several optional cross-cutting addenda, such as the Global and Planetary Health Addendum, which includes One Health and ESH approaches (25). While the addendum partially extends beyond the scope of core curricula, it nevertheless has the potential to guide educators in developing content for both mandatory and elective PHE. In addition to the core curriculum, medical students are required to complete electives from their institutions’ portfolios. Electives provide educators with significant flexibility in curriculum design, often allowing new or emerging topics to be first introduced.

Consequently, PH topics can be integrated into undergraduate medical education by incorporating relevant aspects into existing educational activities (e.g., PH and cardiovascular/respiratory/women’s/child/mental health) or by offering dedicated PH electives. So far, however, no studies have systematically assessed the prevalence or implementation of PHE within medical schools in Germany.

This study therefore aimed to (a) assess the current implementation of PHE in undergraduate medical education in Germany and (b) explore the characteristics of PHE, including educational approaches, learning objectives, interdisciplinarity and student involvement. The results of our study have the potential to guide the development and implementation of high-quality, transformative PHE initiatives, both in Germany and globally.

## 2 Materials and methods

### 2.1 Study design

The study presented in this paper is part of the national mixed-methods *Planetary Health in Medical Education (PlanetMedEd)* Project that aims to comprehensively assess the current state of PHE in medical schools in Germany, including the opportunities and barriers for its implementation in medical curricula (26). The PlanetMedEd Project includes quantitative studies using online surveys and quantitative interviews at medical schools, as well as qualitative interviews with students, educators and deanery staff (Supplementary material 1), which have already been published elsewhere (27, 28). To ensure consistent analysis of PHE across all studies within the PlanetMedEd Project, we developed a *Definition of Planetary Health Topics*, which was used in all studies (Table 2).

Our study employed a sequential design consisting of structured interviews followed by an online survey, both targeting all 39 medical schools that are members of the Association of Faculties of Medicine in Germany (Supplementary material 2). This did not include three newer, partially private medical schools.

**Figure 1:**
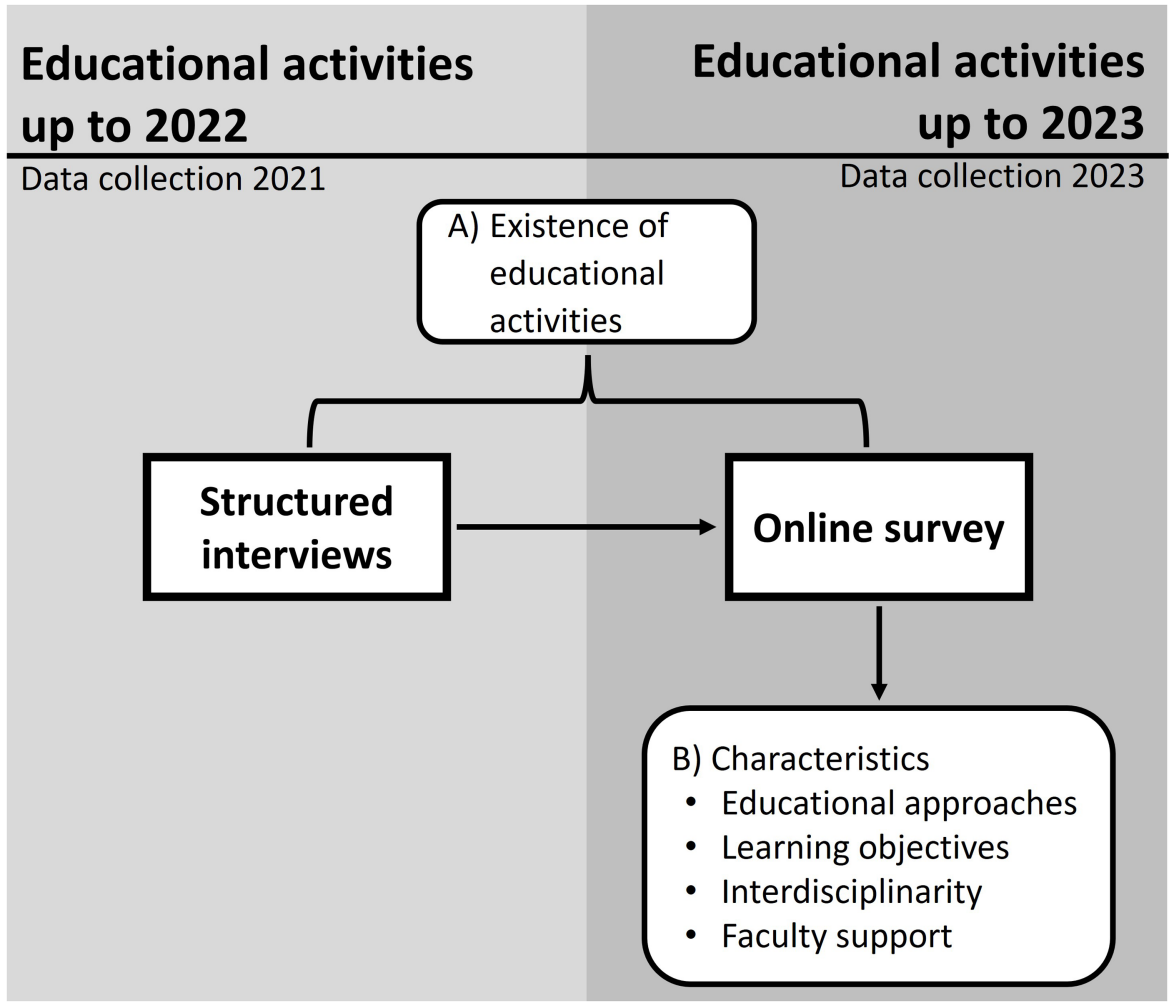
Study design and triangulation of different data sources.

In a first step, we conducted structured interviews to assess the existence of PHE activities offered during the winter semester of 2021/2022 or planned for the summer semester of 2022. Based on these findings, we developed an online survey to assess PHE activities offered during the winter semester of 2022/2023 or planned for the summer semester of 2023, as well as to explore their characteristics in detail. Data from both the interviews and the online survey were integrated to assess the current implementation of PHE (study objective a), whereas the online survey alone was used to explore detailed characteristics (study objective b; Figure 1). Complementary sampling strategies and data collection methods at two different time points were employed to provide a comprehensive overview of PHE. The use of investigator and data triangulation served to enhance the validity of our findings.

### 2.2 Participants and data

#### 2.2.1 Structured interviews

Interview participants were identified in mid-2021 by screening the websites of all medical schools and leveraging existing networks in medical education and PHE in Germany. We invited members from all 39 medical schools in Germany and medical education networks to participate in the interviews. Three different groups (educators, deanery staff, students) were eligible to participate if they could give detailed information about a PHE activity at their respective medical school. Data were included in the study if they covered at least one learning objective from both Chapter 1.1 and Chapter 1.2 of the study’s *Definition of Planetary Health Topics* (Table 2). These chapters refer to foundational knowledge about core areas of PH (e.g. anthropogenic environmental changes; interconnectedness of climate, other anthropogenic environmental changes and health). The guideline and items for the structured interviews were developed by the research team (Supplementary material 3). One researcher (EG) conducted the video interviews between June and September 2021 using Zoom Video Communications without recording. During the interview, this researcher entered the data into a standardized and pretested Microsoft Excel spreadsheet, sharing the screen with the interview partners. Following completion of all interviews, the spreadsheets were sent to the interview partners and the study deaneries of the respective medical school for member checking. In addition, two researchers (EG and EMSS) conducted comprehensive plausibility checks for data consistency.

#### 2.2.2 Online survey

Based on the structured interview guide, we developed the questionnaire for the online survey (Supplementary material 4) between October and December 2022. It underwent qualitative pretesting among six students and quantitative pretesting among five medical doctors and members of the Department of General Practice at the University Hospital Würzburg, who were involved in PHE or experienced in questionnaire development.

The questionnaire was administered in German and covered the following information:

- General information about the medical school
- Characteristics of the educational activity, including learning objectives, teaching and assessment methods
- Cooperation with other educators or students
- Faculty support for Planetary Health

Each participant could report one or two PHE activities per survey completion. For reporting one activity, the questionnaire included 11 open-ended questions and 38 quantitative items, seven of which were mandatory. Reporting a second activity required answering additional six open-ended questions and 30 quantitative items. Participants wishing to fill in more than two educational activities were instructed to restart the survey. In medical schools without PHE, participants were asked five mandatory quantitative items and three open-ended questions. Teaching methods were assessed semiquantitatively using percentage ranges. The survey was implemented in EvaSys (Evasys Survey Automatic Suite, Version 8.0).

The questionnaire was sent to five different groups (Supplementary material 5) in January 2023: (1) The deans of study or study deaneries of all 39 medical schools in Germany and (2) the student councils were contacted via the official email addresses obtained from their respective websites. (3) Local groups affiliated with the *Health For Future (HFF)* movement were identified on the HFF website in 37 cities with medical schools and contacted. (4) To expand outreach through snowball-sampling, invitations were extended to networks including HFF, the *German Climate Change and Health Alliance* (*KLUG e.V.*), and the German *Master of Medical Education*. (5) Participants of the previous structured interviews were also invited to participate in the online survey or to forward the invitation. A first reminder was sent in February 2023, followed by a second reminder in March 2023 to contacts at medical schools that had not yet provided data by then. This sampling strategy aimed to ensure standardized outreach to all 39 medical schools while simultaneously maximizing response rates.

In the structured interviews, students were among the eligible participants. In contrast, the online survey focused on educators and members of (study) deaneries to obtain more detailed data on the educational activities. Students were only eligible if they were actively involved in the PHE activity. All others were encouraged to forward the invitation to eligible participants. For inclusion in the analysis, submitted activities had to cover at least one learning objective of both Chapter 1.1 and Chapter 1.2 of the updated version of our *Definition of Planetary Health Topics* (Table 2).

If participants’ answers did not meet these criteria, two researchers (FG and EMSS) independently assessed those survey responses for eligibility. Any discrepancies were resolved through discussion. Surveys were also screened for duplicate information. Additionally, all data underwent a thorough check for plausibility, with inconsistent entries reviewed by two researchers (FG and EMSS).

### 2.3 Data analysis

Data analysis was performed using IBM SPSS Statistics (Version 27, IBM Corp). Descriptive analyses were conducted for both aspects of the study. Associations between categorical variables in the online survey were assessed with Chi-square tests or Fisher’s exact tests in case of small sample sizes. For significant associations, Cramer’s V was calculated. Associations between research activity and the number of educational activities were investigated using Mann–Whitney-U-tests due to non-normal distribution of the data. Statistical analysis included only courses where corresponding questions were answered yes or no (not unknown).

When qualitative information regarding teaching or assessment methods was provided in the open-ended survey questions that corresponded with quantitative items (“if other, please specify”), it was incorporated in the quantitative analysis. No thematic analysis was performed, as only very limited further information was provided in the open-ended questions. Qualitative data pertaining to the institutional background of the participants was categorized based on curricular subjects defined in the Medical Licensing Regulations.

During data collection, we attempted to distinguish between *courses* defined as stand-alone PHE activities and *classes* defined as PH teaching integrated into other courses. However, this classification proved unhelpful for addressing the main research questions and was not clearly differentiated by the study participants. Consequently, we chose to summarize both stand-alone PH courses and PH teaching sessions incorporated into other courses using the term *educational activities*. They were further categorized based on the number of course units (CU) and stratified by whether they were mandatory or elective.

### 2.4 Ethical considerations

Both the interview study and the online survey received approval by the ethics committee of the University of Würzburg (20210312-01 and 20231123-01). All participants received detailed information about content and conduct of the study as well as data protection measures. The interviewees provided written informed consent to participate in the interviews, which were not recorded. Written informed consent was not required for the survey as only anonymous data were collected.

## 3 Results

In total, 50 individuals participated in the structured interviews, after they had confirmed that their educational activities met the inclusion criteria. In the online survey, 71 individuals entered data, with 66 meeting the inclusion criteria. For six submitted activities, the required learning objectives as inclusion criteria were not reported or marked *unknown*. After thorough evaluation by two researchers (FG and EMSS), three of those were included in the study, based on other clear indicators of PHE, while the remaining three were excluded.

### 3.1 Existence of educational activities

**Figure 2:**
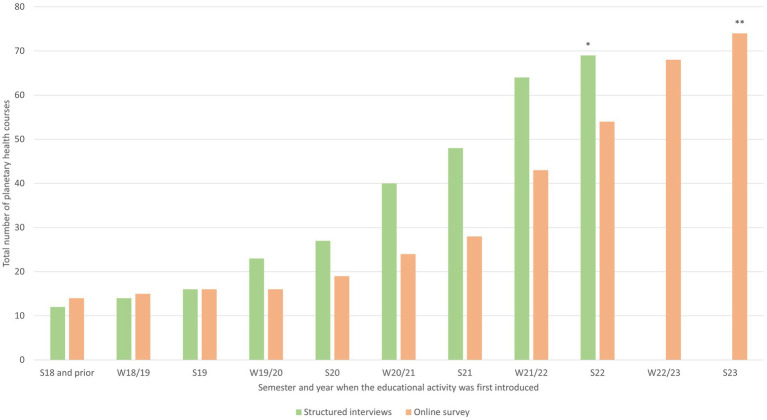
Semester when educational activity was first introduced. *n* = 69 (interviews)/74 (online survey), missing: 26 (interviews)/4 (online survey), *new activities planned for summer semester 2022 (from structured interviews), **new activities planned for summer semester 2023 (from online survey). S, summer semester; W, winter semester.

One hundred and thirty-eight different PHE activities were identified: 60 exclusively through interviews and 43 exclusively through the online survey, 35 of the 138 activities were reported in both. Among medical schools offering PHE, 90% in the interviews and 100% in the online survey offered at least one elective activity. Mandatory activities were offered by 65% of medical schools in the interviews and 40% in the online survey. Triangulated data from both the structured interviews and the online survey are presented in Table 1 and Figure 2. Both the interviews and the online survey indicated an increase in PHE activities with the largest absolute increase observed in the winter semester 2021/2022 (Figure 2).

**Table 1:** Side-by-side display of integrated findings from structured interviews and the online survey, 1 CU = 45 min.

	Structured interviews	Online survey
Contacted medical schools	39	39
Data collection	June–September 2021	January–March 2023
Participating medical schools	31	35
Medical schools that submitted at least one PHE activity	31	30
Integrated findings 1: Interviews and online survey had high response rates. The majority of medical schools in Germany offered at least one Planetary Health educational activity each		
Participants, of which were:	50	66
(Study) deanery	1	9
Students	24	6
Educators (teaching discipline below)^a^	25	51
General practice	··	15
Interdisciplinary subjects^b^	··	11
Occupational medicine	··	6
Internal medicine	··	4
Preclinical subjects	··	3
Pediatrics	··	3
Infectious diseases	··	2
Psychiatry	··	2
Hygiene, microbiology, virology	··	1
Pharmacology, toxicology	··	1
Medical education	··	1
Medical history, theory, ethics	··	1
No answer	··	1
Integrated findings 2: Students and educators participated almost equally frequent in the interviews. The online survey primarily targeted educators from diverse professional backgrounds to investigate more education characteristics in-depth		
Activities identified only in this study part	60	43
Activities identified in both study parts	35	35
Total number of submitted activities	95	78
Median number per medical school	2	2
Range per medical school	1–9	1–8
Mandatory vs. elective educational activities	*n* = 95	*n* = 78
Mandatory	32 (34%)	24 (31%)
Elective	63 (66%)	54 (69%)
Integrated findings 3: A median of two activities per medical school was identified. The majority of reported activities were not mandatory, but elective activities catering to a limited proportion of students		
Course units (CU): Mandatory	*n* = 27	*n* = 24
Median	2 CU	2 CU
Range	1–7 CU	1–8 CU
Course units (CU): Elective	*n* = 40	*n* = 53
Median	9 CU	16 CU
Range	1–45 CU	1 – over 60 CU
Integrated findings 4: Mandatory educational activities reported were typically single lessons (2 CU = 90 min), while electives generally comprised more course units		

Data of the structured interviews includes data retrieved from one written feedback by a deanery. ^a^Data about the field were only obtained in the online survey. ^b^Interdisciplinary subjects: include participants from institutes whose teaching is part of the interdisciplinary subjects as defined by the Medical Licensing Regulations (1: epidemiology, medical biometry, and health informatics. 3: health economics, health system and public health. 6: clinical environmental medicine. 10: prevention and health promotion). CU, course unit; PH, planetary health.

### 3.2 Characteristics of educational activities

In total, 78 activities from the online survey were included in the analyses of detailed characteristics. Few activities lacking specific information for a particular question were excluded from the analysis pertaining to that question, as shown by the indicated *n* for the respective question.

**Figure 3:**
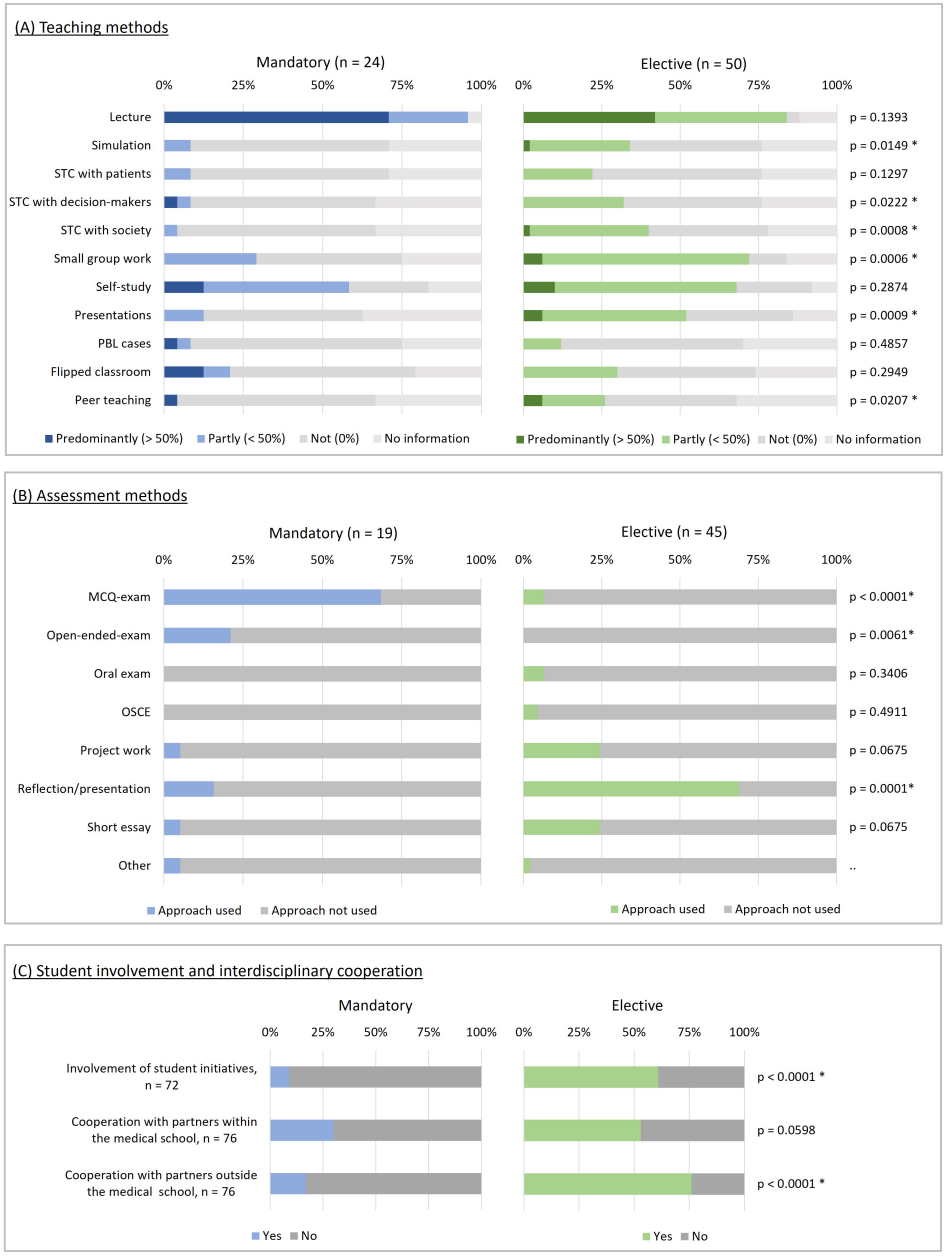
Characteristics of educational activities. Left: Mandatory, right: elective, multiple selection possible, * *p* < 0.05. **(A)** Teaching methods, PBL, problem-based learning; STC, skills training for communication. **(B)** Assessment methods, MCQ, multiple choice quiz; OSCE, objective structured clinical examination. **(C)** Student involvement and interdisciplinary cooperation.

The number of participants varied, ranging from ≤10 to >101, with a median of >101 participants for mandatory and 11–20 participants for elective activities. Educational approaches differed between mandatory and elective activities as illustrated in Figures 3A and 3B. Mandatory activities predominantly used lectures as a teaching method and employed multiple choice quizzes (MCQ) or open-ended-text exams as assessment methods. Electives used a more varied set of teaching and assessment methods aiming at upper Miller competency levels (“Shows How,” “Does”) such as simulation, skills training for communication (STC) or small group work as teaching methods and final reflection or project work as assessment methods.

Most learning objectives (LO) were reported more frequently for elective as compared to mandatory educational activities (Table 2). Notably, LOs related to transformative competencies (Chapter 3) were significantly more emphasized in elective activities than in mandatory ones.

**Table 2:** Learning objectives addressed in mandatory and elective educational activities, multiple selection possible.

Learning objective (LO)	Mandatory educational activities	Elective educational activities
	Percentages represent the proportion of activities reporting this LO among all activities with available data on the respective chapter	
Chapter 1. Graduates demonstrate foundational knowledge about core areas of Planetary Health. They demonstrate understanding of associations regarding the following topics:		
1.1: Anthropogenic environmental changes, e.g.: (*n* = 76: 22 mandatory, 54 elective), (= inclusion criteria)		
Climate change	75%	98%
Other environmental changes	38%	59%
Planetary boundaries	50%	85%
Systems research	42%	67%
At least one of these LO	100%	100%
Chapter 1.2: Interconnectedness of climate, other anthropogenic environmental changes and health, e.g.: (*n* = 77: 24 mandatory, 53 elective), (= inclusion criteria)		
Heat	79%	92%
Other extreme weather events	46%	58%
Infectious diseases	50%	75%
Toxin-mediated diseases	21%	34%
Cardiovascular diseases	46%	60%
Endocrinological diseases^a^	21%	21%
Oncological diseases^a^	17%	26%
Allergies	54%	74%
Maternal and child health	21%	47%
Neurological diseases	13%	15%
Mental health	42%	70%
Migration and violent conflicts	38%	53%
Connections of nutrition, environment and health	38%	79%
Co-benefits^b^	42%	74%
At least one of these LO	100%	100%
Chapter 1.3: Populations that are particularly affected by global environmental changes and their vulnerability factors (*n* = 69: 21 mandatory, 48 elective) For example: Children, infants, elderly, people requiring care, (pregnant) women, people with pre-existing conditions, polypharmacy, pre-existing psychological vulnerability, low socio-economic status, precarious living/working conditions, migration background, populations in regions without sufficient social security systems, climate justice (incl. global and local discrepancy between those responsible and those affected)		
At least one of these LO (*p* = 0.1609)	86%	96%
Chapter 2: Graduates reflect on their responsibility to establish, maintain and foster human health and the natural and social systems on which it depends (*n* = 73: 22 mandatory, 51 elective) For example: sustainable transformation of all relevant areas of society including mobility, nutrition, energy, agriculture, consumption, economy, healthcare, social and legal norms		
At least one of these LO (*p* = 0.4094)	95%	90%
Chapter 3: Graduates describe and demonstrate skills to stimulate and implement transformative change. They know concrete examples of transformative action and can implement measures themselves, e.g.: (*n* = 70: 21 mandatory, 49 elective)		
Climate communication	43%	73%
Science communication	5%	41%
Transdisciplinary collaboration	24%	65%
Project management	5%	33%
Sustainable healthcare	57%	88%
Working with heat action plans	19%	41%
None	29%	6%
At least one of these LO (*p* = 0.0176*, Cramer’s V: 0.3073)	71%	94%

LO, learning objective. *Statistically significant differences: *p* < 0.05. ^a^These LOs were added to this table after constructive feedback for the online survey and were not part of the definition during the structured interviews. ^b^This LO was moved from Chapter 3 to Chapter 1.2 to introduce co-benefits on a knowledge level. During the structured interviews, it was still listed under Chapter 3. This list of learning objectives reflects the definition of Planetary Health used within the PlanetMedEd Project as previously published (28). It was based on the German National Competency-based Learning Objectives Catalog in Medicine (NKLM 2.0), specifically on the optional addendum Planetary and Global Health (25).

Student initiatives or partners from other disciplines were involved in 32 activities. Almost a quarter (*n* = 18) were open to non-medical students within the medical school or other faculties. Student involvement and collaboration with other partners were more frequent in electives (Figure 3C). When student initiatives were involved, they were actively engaged in initiating (72%), planning, or implementing (both 75%). Educational activities involving student initiatives aimed at transformative LOs (Chapter 3) significantly more often than those without student involvement (100% vs. 74%, *n* = 65, *p* = 0.002, Cramer’s-V = 0.38). As transformative LOs were predominantly covered in electives, these were analyzed separately to mitigate potential confounding. In the stratified analysis, transformative LOs were significantly more often covered in electives with student involvement (*p* = 0.04, Cramer’s-V = 0.35). No significant differences were found for LOs from Chapter 1.3 and Chapter 2 of the *Definition of Planetary Health Topics*.

Research focused on PH was reported for 19 medical schools, accounting for 54% of the participating and 49% of all medical schools in Germany. Among others, this included a chair for climate change and health and different working groups with a focus on PH, for example at departments for hygiene, infectious diseases or general practice. Medical schools without research activity on PH reported a median of one PHE activity, whereas those with such research activity reported a median of three. This difference was statistically significant (U = 56.5, Z = −2.38, *p* = 0.009). Participants who submitted at least one activity, were also asked about the presence of a dedicated PHE coordinator at their medical school, which was reported by eight schools.

## 4 Discussion

To our knowledge, this is the first nationwide study to thoroughly investigate the current implementation and characteristics of PHE in undergraduate medical education across all medical schools with this level of detailed overview of educational approaches, learning objectives, student involvement and interdisciplinarity. Our study holds transformative potential as suggested by previous research on PHE (17). High response rates of 80 and 90% of medical schools in the interviews and the online survey, respectively, provide a solid foundation for a granular assessment of PHE in Germany, highlighting opportunities for curriculum innovation in light of the Ten Characteristics of High-quality PHE developed in a companion study of the PlanetMedEd Project (hereinafter referred to as Ten Characteristics) (27).

### 4.1 Existence of educational activities

Only 35 of all 138 activities were reported in both interviews and the online survey, with the majority appearing only in one of the two. This highlights the value of combining different research methods to obtain a comprehensive overview. We observed a continuous increase in PHE activities since 2019. Educators from diverse professional backgrounds contributed data to the study, indicating the involvement of multiple disciplines in PHE.

Most medical schools offered at least one PHE activity. Only one-third of all activities, however, were mandatory, highlighting the need for greater curricular integration (27). Education predominantly offered in elective format risks reaching only students already interested in the field. Moreover, integrating PHE into existing mandatory education appears to be preferred by students (29), suggesting potential for strong coalitions with educators aiming to strengthen PHE. With a median of two course units, mandatory educational activities mainly seemed to follow a single-session approach, limiting the coverage of complex PH topics that go beyond an introduction to the field (30).

### 4.2 Characteristics of educational activities

PHE requires transformative educational methods (6, 31). Lectures, however, were still the predominant teaching method in both elective and mandatory activities. Innovative educational approaches including problem based learning (PBL), simulations, skills training, and peer teaching were more common in electives. These therefore addressed the need for transformative educational methods in PHE more comprehensively (6, 31). Assessment methods targeting higher levels of Miller’s competency framework (22), such as project work, final reflections, or presentations were also reported more frequently for elective educational activities. Objective Structured Clinical Examinations (OSCE) were almost non-existent, despite recent research indicating their importance in PHE (13). Thus, we found different “innovative and proven didactic methods,” from the Ten Characteristics (27) mainly in electives. Combining different innovative educational approaches seems most appropriate to address the complexity of PHE (27, 30) and may contribute to better learning outcomes (32).

Knowledge, values and competencies – key areas of PHE (6, 8) – reflected in Chapters 1–3 of our *Definition of Planetary Health Topics* (Table 2) were addressed to varying extents. All educational activities addressed LOs related to knowledge, although the depth of coverage varied (Chapter 1). Comprehensive integration of PHE into medical curricula, however, requires broad coverage of all relevant topics, including the “responsibility of health professionals” and “transformative competencies” (27). LOs related to responsibility (Chapter 2) were covered in nearly all educational activities, while transformative competencies (Chapter 3) were primarily addressed in elective courses. The broader scope of learning objectives covered in electives may reflect the higher number of course units in these courses. To fully leverage the potential of PHE, educators should place greater emphasis on integrating content that strengthens transformative competencies of students.

Partnership with students can enrich PHE (6) and is described as an important quality characteristic (27). In our study, student involvement in teaching activities was predominantly reported for electives and was significantly associated with transformative learning objectives in our study, underscoring the crucial role of students in PHE. Despite the recognized importance of inter- and transdisciplinarity in PHE (6, 27), interdisciplinary collaborations with academic partners, both within and outside the medical school, were reported for only about half of the educational activities assessed, with electives being particularly prominent. Teaching methods with the potential to advance transdisciplinarity, such as skills training for communication with the general public, were almost exclusively reported for electives.

### 4.3 Guiding Planetary Health Education

Research focused on PH was reported for approximately half of the medical schools. Detailed questions about the structure and content of research activities were not included in this study, as the focus was on educational activities. The positive association between PH research and the number of PHE activities suggests that research may play a key role in driving the integration of PHE into medical curricula. Potential underlying mechanisms for this association include increased awareness, broader faculty support, or the availability of experts capable of delivering dedicated PH teaching.

PHE coordinators could help address key barriers to the inclusion of PHE in medical curricula, such as lack of faculty support, time, expertise or confidence (30). Given that PHE coordinators were reported in only eight medical schools, establishing coordinators should be considered a priority for the development of PHE.

The lack of academic requirements and binding standards for PHE (30), including the non-mandatory PH addendum to the National Competency-based Learning Objectives Catalog in Medicine (25), hinders curriculum innovation in Germany and may contribute to the heterogeneous implementation of PHE observed in this study. These findings emphasize the need for comprehensive integration of PH content into mandatory curricula through an intersectional approach. PH aspects should be incorporated into various subjects, their assessments, and the state exams for the medical degree. Additionally, elective courses offer opportunities to provide more tailored and more comprehensive content for students particularly interested in PH (33). Our *Definition of Planetary Health Topics* (Table 2) outlines numerous opportunities to integrate PH across different subjects. At the same time, multiplier training within medical education networks could enhance the didactic skills of educators. Established elective courses can guide curriculum innovation as best practice examples, often meeting criteria for high-quality PHE (27). Consensus recommendations developed by medical associations, based on published learning objectives, can support efforts to mainstream PHE in undergraduate medical education (25) and help to increase the significance that educators attribute to PH, a known lever for successful PHE implementation (30).

### 4.4 Limitations

Several factors potentially limit the comprehensiveness of the PHE overview presented in this study. First, the sampling strategy involving PH networks may introduce selection bias. To attenuate this bias, we also invited the (study) deaneries of all 39 medical schools. Second, educational activities consisting of only one course unit might be underrepresented, as these activities are often not included in syllabi and related official documents. We aimed to reduce underreporting by combining structured interviews with an online survey and involved students, educators and study deaneries. Third, given the study’s focus on PH stand-alone courses or entire PH sessions, PH content integrated into established educational formats at a micro level (e.g., integration of one slide focused on PH) was not captured. Fourth, as many study participants represented their medical school in an official capacity, social desirability bias might have induced an overestimation of addressed learning objectives and PH content. We anticipate that the interview study had a greater impact in this regard compared to the anonymous online survey. Fifth, as the study team was based in the general practice department, there may be an overrepresentation of educational activities related to general practice due to selection bias. Finally, underreporting might have occurred because the survey completion time amounted to approximately 15 minutes for one educational activity and 25 minutes for two. The comprehensive nature of our questionnaire, however, allowed us to explore various aspects of teaching activities in detail.

## 5 Conclusion

Our findings bridge the gap between numerous calls for action and frameworks for PHE and the lack of a comprehensive overview of PHE for the 39 assessed medical schools in Germany. While many medical schools now offer PH elective courses, comprehensive mandatory educational activities meeting standards for high-quality PHE remain scarce. Amidst ongoing and future reforms of medical curricula, well-established elective courses can serve as best-practice examples to guide the broader integration of PHE. This study can serve as a blueprint for monitoring curriculum innovation in Germany and internationally, advocating for the integration of additional PHE content by highlighting both the current state and existing implementation gaps in curricular PHE.

## Data Availability

The raw data supporting the conclusions of this article will be made available by the authors, without undue reservation.
