# Supplementary material for: Planetary health education in undergraduate medical education in Germany: results from structured interviews and an online survey within the national PlanetMedEd Project
Source: Front Med (Lausanne). 2025 Feb 11;11:1507515. doi: 10.3389/fmed.2024.1507515 (PMC11851124; doi:10.3389/fmed.2024.1507515)
Supplement: Supplementary file 1 [file Data_Sheet_1.pdf]

Supplementary Material

Planetary Health Education in undergraduate medical education in Germany: Results from structured interviews and an online survey within the national PlanetMedEd Project

Table of Contents

1 PlanetMedEd- Study design 2

2 Contacted faculties 3

3 Guide for structured interviews 4

4 Questionnaire online survey (original German version, English translation see below) 10

5 Sampling Strategy (Online Survey) 28

1 PlanetMedEd- Study design

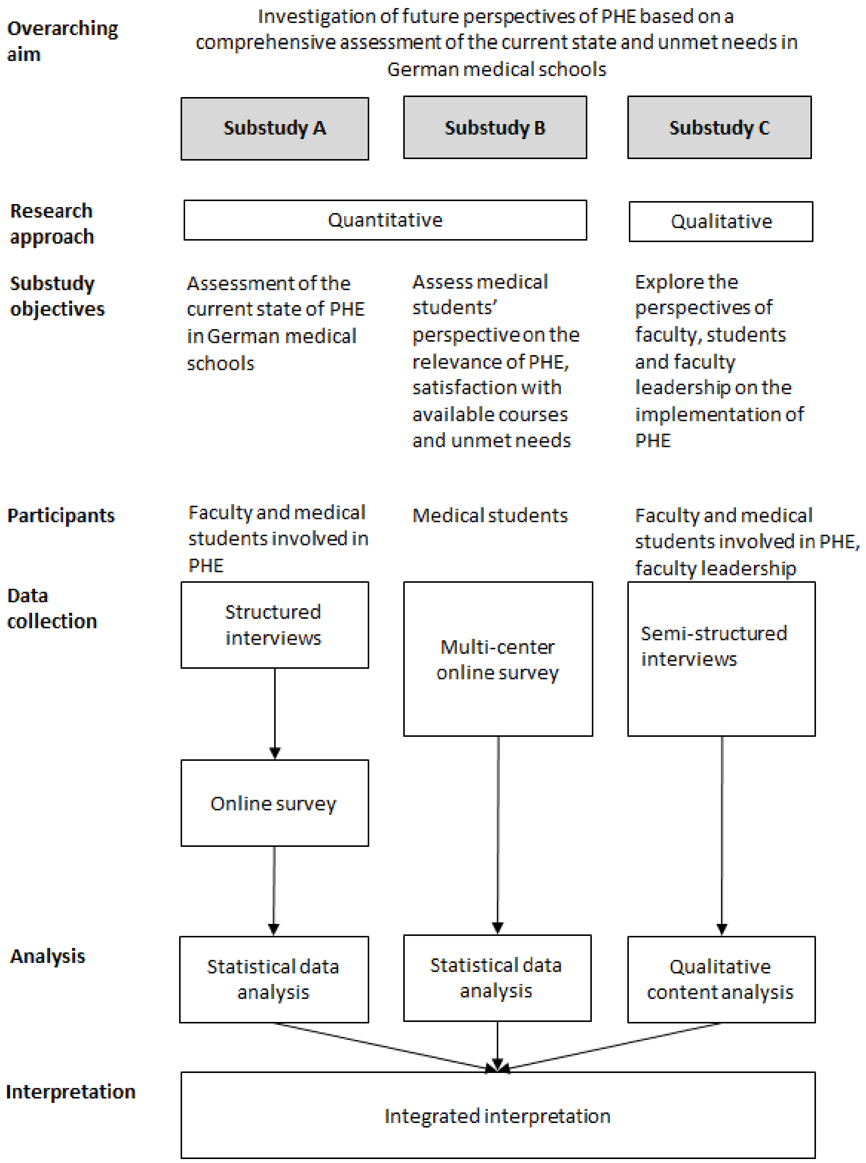

Supplementary Figure 1. PlanetMedEd – Study design. This is an updated version of the already published study design by Simon et al.

Simon J, Parisi S, Wabnitz K, Simmenroth A, Schwienhorst-Stich E-M. Ten characteristics of high-quality planetary health education-Results from a qualitative study with educators, students as educators and study deans at medical schools in Germany. Front Public Health (2023) 11:1143751. doi:10.3389/fpubh.2023.1143751

2 Contacted faculties

- Aachen
- Augsburg
- Berlin
- Bielefeld
- Bochum
- Bonn
- Dresden
- Duisburg
- Düsseldorf
- Erlangen
- Frankfurt
- Freiburg
- Gießen
- Göttingen
- Greifswald
- Halle
- Hamburg
- Hannover
- Heidelberg
- Homburg-Saar
- Jena
- Kiel
- Köln
- Leipzig
- Lübeck
- Magdeburg
- Mainz
- Mannheim
- Marburg
- München LMU
- München TU
- Münster
- Oldenburg
- Regensburg
- Rostock
- Tübingen
- Ulm
- Witten-Herdecke
- Würzburg

3 Guide for structured interviews

This is an English translation of the interview guide, the original interview guide was in German, the structured interviews were also held in German.

**Introduction**	
Welcome	Welcome to this short interview. We are grateful that you are taking part in our study.
Explain the objectives of the study	With the help of these interviews, we want to find out more about the current situation of planetary health education at medical schools in Germany. Therefore, we would like to provide a structured overview of existing and planned courses at all medical schools.
Time frame	The interview will last approximately 15 minutes.
Voluntariness, confidentiality	As described in the consent form, your participation is voluntary. You can withdraw from the interview at any moment. The interview will not be recorded. Your responses will be documented in a checklist.
Declaration of consent	You did already provide us with your signed consent form.
Do you have any questions so far? We will now begin with the interview.	

**Main part of the interview**	*Displayed here are the options in the spreadsheet to fill in the information, which was jointly viewed by the interview participant and the researcher*
At which medical school do you work/study?	
We have sent you our definition of planetary health education via email. Here you can see the list again. If at least one aspect from 1.1. and one aspect from 1.2. is taught, the course will be included in the study.	*Shared screen: "Definition of planetary health education"*
Environmental medicine is part of the curriculum at medical schools. Is planetary health taught as part of	yes/ no / unknown

the environmental medicine course at your medical school?	
If so, how large is the share of planetary health in environmental medicine teaching overall?	xx course units of xx course units, otherwise share (%) of whole course
Is there a global health teaching offer at your medical school? If so, is planetary health part of the global health teaching offer?	Yes/no/unknown
Is the share of PH in global health quantifiable?	Yes/no/unknown
If so, how large is the share of planetary health in global health teaching overall?	xx course units of xx course units, otherwise share (%) of whole course
Is there a planetary health course?	yes/ no / unknown
What is the title of the course?<br><br>☐ *Let's assess each title individually below.*	
Are there specific learning objectives?	yes/ no / unknown
Open-text learning objectives (*if possible, may we request a document including all learning objectives via email)*	
We now assess the topics that are covered in the course. For this part, I will share the screen with the learning objectives which are part of planetary health education according to the NKLM [German National Competency-based Learning Objectives Catalogue in Medicine]	*Shared screen: "Definition of planetary health education for the study"*
Which topics from 1.1 and 1.2 are covered in the course?	1.1<br>climate change<br>other environmental changes<br>planetary boundaries<br>systems research<br>1.2<br>heat<br>other extreme weather events<br>infectious diseases<br>toxin-mediated diseases<br>cardiovascular diseases<br>allergies<br>maternal and child health

	neurological diseases<br>mental health<br>migration and violent conflicts<br>connections of nutrition, environment and health
Are there any topics taught from chapter 1.3?	yes/ no / unknown
Are there any topics taught from chapter 2?	yes/ no / unknown
If so, which topics ?	co-benefits<br>mobility<br>nutrition<br>energy<br>agriculture<br>consumption<br>economy<br>health care<br>social/legal values and norms<br>unknown
Are there any topics taught from chapter 3 ?	yes/ no / unknown
If so, which topics ?	climate communication<br>science communication<br>transdisciplinary collaboration<br>project management<br>sustainable healthcare<br>working with heat action plans<br>unknown
Where is your course located in the curriculum?	curricular (except for environmental medici[illegible]<br>environmental medicine<br>elective (clinical study part)<br>elective (preclinical study part)<br>elective (both)<br>extracurricular<br>If other, please explain in open-text field
Are there any student initiatives involved in the course?	yes/ no / unknown
If so, in which way ?	- initiation<br>- planning<br>- implementation

<table>
<tr><td>If so, which initiatives?</td><td>Free text field</td></tr>
<tr><td>When was the course first introduced?</td><td>winter semester / summer semester □ year</td></tr>
<tr><td>Are there new courses planned in the future?</td><td>yes/ no / unknown</td></tr>
<tr><td>Which is the target group for this course?</td><td>preclinical students (1st - 4th semester)
clinical students (5th - 10th semester)
all semesters
Interdisciplinary target group</td></tr>
<tr><td>What is the average number of participants?</td><td>5 to 10
11 to 20
21 to 30
31 to 40
41 to 50
> 50</td></tr>
<tr><td>We will now assess the teaching methods for each course. We will also ask you to state the approximate proportion of the teaching format:</td><td></td></tr>
<tr><td>Lecture</td><td rowspan="6">- mostly (more than 50%)
- partially (less than 50%)
- not at all (0%)</td></tr>
<tr><td>Simulation</td></tr>
<tr><td>Skills training for communication (Conversations with patients)</td></tr>
<tr><td>Skills training for communication (conversations with decision-makers)</td></tr>
<tr><td>Skills training for communication (Conversations with Society)</td></tr>
<tr><td>Small group work</td></tr>
</table>

Self-study	
Problem-based-learning	
Using of prepared teaching materials	
Peer teaching	
Which teaching formats do you choose to address the learning objectives from 2.) and 3.), i.e. methods for transformative approaches?	
Which examination method is used?	mc-exam<br>open-text-exam<br>oral exam<br>OSCE<br>project work<br>final reflection/presentation<br>short essay<br>participation<br>other
Is there an evaluation of the course?	yes/ no / unknown
Is there any accompanying research for the course?	yes/ no / unknown
Are cooperation partners outside the medical school involved in the course?	yes/ no / unknown
If so, which?	
How many students are there at your medical school per semester?	
Are there **other courses** that address planetary health issues?	
**Final questions:**	
Comprehensiveness of the information: Were you able to provide a comprehensive overview of the courses offered at your location?	

Are there any other people we should contact to get a comprehensive overview of the courses offered at your location?	
Do you agree with the publication of the results? No personal data will be published; only information about the courses, linked to the respective location of the medical school.	yes/no
Thank you and goodbye.	

4 Questionnaire online survey (original German version, English translation see below)

MUSTER

EvaSys	Lehrangebote zu Planetarer Gesundheit	Electric Paper

Medizinische Fakultät Würzburg — Dr. med. Eva-Maria Schwienhorst-Stich

AG Klima und Planetare Gesundheit — PlanetMedEd-Studie: Lehre zu Planetarer

Bitte so markieren: ☐ ☒ ☐ ☐ ☐ Bitte verwenden Sie einen Kugelschreiber oder nicht zu starken Filzstift. Dieser Fragebogen wird maschinell erfasst.

Korrektur: ☐ ■ ☐ ☒ ☐ Bitte beachten Sie im Interesse einer optimalen Datenerfassung die links gegebenen Hinweise beim Ausfüllen.

1. Vorbemerkung

Sehr geehrte Teilnehmende,

die Arbeitsgruppe „Klima und Planetare Gesundheit" der Medizinischen Fakultät Würzburg möchte im Rahmen der PlanetMedEd-Studie mit Ihrer Hilfe eine Bestandsaufnahme zu Lehr- und Lernaktivitäten auf dem Gebiet der **Planetaren Gesundheit (Klima, Umwelt, Gesundheit)** erheben. Der gegenwärtige Stand soll eine Übersicht liefern und damit eine wichtige Orientierungshilfe bei der (Weiter-)Entwicklung von Lehrveranstaltungen bieten.

Die Beantwortung der Fragen nimmt ca. **15 Minuten** in Anspruch. Falls es **keine entsprechenden Angebote** an Ihrem Standort gibt, so teilen Sie uns dies bitte ebenfalls kurz im Fragebogen mit. Die Bearbeitungszeit beträgt in diesem Fall nur ca. **2 Minuten**.

Gerne können Sie in dieser Umfrage verschiedene Lehrveranstaltungen beschreiben. Bitte beantworten Sie hierfür die Fragen **zunächst für eine der Lehrveranstaltungen**. Anschließend können Sie die entsprechenden Fragen für eine zweite Veranstaltung beantworten. Hierfür benötigen Sie zusätzlich ca. 10 Minuten.

Bei Rückfragen wenden Sie sich bitte an Dr. med. Eva-Maria Schwienhorst-Stich (Schwienhor_E@ukw.de) oder den Doktoranden Fabio Grieco (fabio.grieco@stud-mail.uni-wuerzburg.de).

Für Ihre Mitarbeit bedanken wir uns bereits im Vorfeld recht herzlich!
Für einen reibungslosen Ablauf sollten Pop-Up Blocker in Ihrem Browser deaktiviert sein.

2. Datenschutzrechtliche Hinweise

Nachfolgend finden Sie Informationen zum Datenschutz bei dieser Studie. Ihr Einverständnis ist Voraussetzung für die Teilnahme an der Studie.
Die Daten der Umfrage werden **anonymisiert** erhoben. Mit Ihrer Teilnahme stimmen Sie der Speicherung und Auswertung der Daten für Forschungszwecke sowie einer etwaigen Publikation der Daten zu. Ihre **Teilnahme** an der Studie ist **freiwillig** und kann zu jedem Zeitpunkt abgebrochen werden.
Mit Ihrer Teilnahme stimmen Sie den datenschutzrechtlichen Hinweisen zu.

Pflichtangaben gemäß § 13 Datenschutzgrundverordnung (DSGVO):

Verantwortliche für die Befragung ist die Julius-Maximilians-Universität Würzburg, Sanderring 2, 97070 Würzburg, Tel.: 0931/31-0 , info@uni-wuerzburg.de
Der Behördliche Datenschutzbeauftragte ist der Datenschutzbeauftragte der Universität Würzburg, Sanderring 2, 97070 Würzburg ,Tel.: 0931/31-0, datenschutz@uni-wuerzburg.de

Zweck der Verarbeitung ist die Evaluation bestehender Lehrangebote zu Planetarer Gesundheit an medizinischen Fakultäten in Deutschland.

Rechtsgrundlage der Verarbeitung ist Art. 6 Abs. 1 DSGVO.
Berechtigtes Interesse gemäß Art. 6 Abs. 1 DSGVO: Forschung im Bereich medizinischer Ausbildung (Lehre zu Planetarer Gesundheit).
Die Umfrage erfolgt anonym, es werden **keine personenbezogenen Daten** erhoben.
Die Daten werden lediglich an Beteiligte an der Studie übermittelt, jedoch nicht an Drittländer oder internationale Organisationen.

Wir informieren darüber, dass eine Identifikation der Befragten nicht vollständig ausgeschlossen werden kann, aber nicht beabsichtigt ist und nicht systematisch betrieben wird.
Eine Löschung der Angaben nach Absenden ist aufgrund der Anonymität der Daten nicht möglich.

MUSTER

2. Datenschutzrechtliche Hinweise [Fortsetzung]

2.1 Ich habe die obigen Informationen und Hinweise gelesen, hatte ausreichend Zeit, mich für die Teilnahme zu entscheiden und willige in die Teilnahme ein.
☐ Ja

3. Allgemeine Informationen

3.1 Für welche medizinische Fakultät (Standort) beantworten Sie diesen Fragebogen?

☐ Aachen	☐ Augsburg	☐ Berlin
☐ Bielefeld	☐ Bochum	☐ Bonn
☐ Dresden	☐ Duisburg-Essen	☐ Düsseldorf
☐ Erlangen	☐ Frankfurt	☐ Freiburg
☐ Gießen	☐ Göttingen	☐ Greifswald
☐ Halle-Wittenberg	☐ Hamburg	☐ Hannover
☐ Heidelberg	☐ Homburg	☐ Jena
☐ Kiel	☐ Köln	☐ Leipzig
☐ Lübeck	☐ Magdeburg	☐ Mainz
☐ Mannheim	☐ Marburg	☐ München LMU
☐ München TU	☐ Münster	☐ Oldenburg
☐ Regensburg	☐ Rostock	☐ Tübingen
☐ Ulm	☐ Witten/Herdecke	☐ Würzburg

3.2 In welcher Organisationseinheit bzw. in welcher Funktion arbeiten Sie an der medizinischen Fakultät?
☐ Dekanat, Studiendekanat ☐ Dozierende ☐ Sonstiges

3.3 Wenn Dozierende, an welcher Klinik/ an welchem Institut/ für welches Fach?

3.4 Wenn Sonstiges, bitte weiter spezifizieren (z.B.: Klinik/Institut, genaue Funktion)

3.5 Gibt es an Ihrer Fakultät bis einschließlich Wintersemester 22/23 Lehrveranstaltungen zu Planetarer Gesundheit oder sind solche für das Sommersemester 23 geplant?

Hier finden Sie eine Definition zu Lehre in Planetarer Gesundheit. In die Befragung werden Veranstaltungen eingeschlossen, wenn jeweils mindestens ein Aspekt aus 1.1. UND 1.2. gelehrt wird.
☐ Ja ☐ Nein

3.6 Gibt es an Ihrer Fakultät ein Institut, eine Abteilung oder eine Arbeitsgruppe für **Forschung** im Bereich Planetare Gesundheit?
☐ Ja ☐ Nein ☐ Keine Angabe

3.7 Wenn ja, was genau?

4. Grunddaten Veranstaltung 1

4. Grunddaten Veranstaltung 1 [Fortsetzung]

4.1 Titel der Veranstaltung

Bitte wählen Sie zunächst nur eine Veranstaltung aus. Anschließend haben Sie die Möglichkeit, die Fragen für eine zweite Lehrveranstaltung zu beantworten.

4.2 Wie viele Unterrichtseinheiten umfasst die Veranstaltung im gesamten Semester? (1 UE = 45 min)

☐ 1	☐ 2	☐ 3
☐ 4	☐ 5	☐ 6
☐ 7	☐ 8	☐ 9
☐ 10	☐ 11	☐ 12
☐ 13	☐ 14	☐ 15
☐ 16	☐ 17	☐ 18
☐ 19	☐ 20	☐ 21
☐ 22	☐ 23	☐ 24
☐ 25	☐ 26	☐ 27
☐ 28	☐ 29	☐ 30
☐ 31	☐ 32	☐ 33
☐ 34	☐ 35	☐ 36
☐ 37	☐ 38	☐ 39
☐ 40	☐ 41	☐ 42
☐ 43	☐ 44	☐ 45
☐ 46	☐ 47	☐ 48
☐ 49	☐ 50	☐ 51
☐ 52	☐ 53	☐ 54
☐ 55	☐ 56	☐ 57
☐ 58	☐ 59	☐ 60
☐ > 60	☐ Keine Angabe	

4.3 Seit wann besteht die Lehrveranstaltung in ihrer aktuellen Form?

☐ vor WS17/18	☐ WS17/18	☐ SS18
☐ WS18/19	☐ SS19	☐ WS19/20
☐ SS20	☐ WS20/21	☐ SS21
☐ WS21/22	☐ SS22	☐ WS22/23
☐ SS23 (geplant)	☐ Unbekannt	

4.4 Gab es in der Vergangenheit eine Vorgängerveranstaltung mit anderem Aufbau oder anderen Inhalten? ☐ Ja ☐ Nein

4.5 Wenn ja, seit wann bestand diese Vorgängerveranstaltung?

☐ vor WS17/18	☐ WS17/18	☐ SS18
☐ WS18/19	☐ SS19	☐ WS19/20
☐ SS20	☐ WS20/21	☐ SS21
☐ WS21/22	☐ SS22	☐ Unbekannt

*Bitte beachten Sie: Die nachfolgenden Fragen beziehen sich ausschließlich auf die **Veranstaltung in ihrer neuesten Form**.*

4.6 Welches Format hat die Lehrveranstaltung? ☐ Gesamter Kurs ("Course") ☐ Einzelveranstal-tung(en) innerhalb eines Kurses ("Class")

4. Grunddaten Veranstaltung 1 [Fortsetzung]

4.7 Falls es sich um (eine) einzelne Veranstaltung(en) innerhalb eines Kurses handelt, wie groß ist in etwa der Anteil am gesamten Kurs in Prozent?
- ☐ 0-20%
- ☐ 21-40%
- ☐ 41-60%
- ☐ 61-80%
- ☐ 81-100%
- ☐ keine Angabe

4.8 Wo ist die Veranstaltung im Curriculum verortet? (Mehrfachauswahl möglich)
- ☐ Curricular, Vorklinik
- ☐ Curricular, Klinik
- ☐ Wahlpflichtfach, Vorklinik
- ☐ Wahlpflichtfach, Klinik
- ☐ Extracurricular
- ☐ Bestandteil des Fachs "Umweltmedizin"
- ☐ Veranstaltung offen für Studierende weiterer medizinischer Fächer an der medizinischen Fakultät (z.B.: Zahnmedizin, Biomedizin, Tiermedizin)
- ☐ Veranstaltung offen für Studierende anderer Fakultäten

4.9 Wie hoch ist die Teilnehmendenzahl der Lehrveranstaltung im aktuellen Semester? (bzw. geplante Zahl, falls Beginn erst SS23)
- ☐ ≤ 10
- ☐ 11-20
- ☐ 21-30
- ☐ 31-40
- ☐ 41-50
- ☐ 51-60
- ☐ 61-70
- ☐ 71-80
- ☐ 81-90
- ☐ 91-100
- ☐ > 101
- ☐ Keine Angabe

4.10 Hier ist Platz für Ihre Anmerkungen zur Teilnehmendenzahl (z.B. Entwicklungen, Schwankungen, Unsicherheiten)

☐

4.11 Welche Prüfungsmethode(n) kommt/kommen zur Anwendung? (Mehrfachauswahl möglich)
- ☐ Klausur (Multiple Choice)
- ☐ Klausur (Freitext)
- ☐ Mündliche Prüfung
- ☐ OSCE (Objective Structured Clinical Examination)
- ☐ Projektarbeit
- ☐ Abschlussreflexion/Präsentation
- ☐ Kurzaufsatz (Short Essay)
- ☐ Regelmäßige Teilnahme
- ☐ Unbekannt
- ☐ Sonstiges

4.12 Wenn Sonstiges, bitte Prüfungsmethode(n) ergänzen:

☐

5. Kooperationen Veranstaltung 1

5.1 Ist an dem Lehrangebot eine studentische Initiative beteiligt?
- ☐ Ja
- ☐ Nein
- ☐ Unbekannt

5.2 Wenn ja, auf welche Weise? (Mehrfachauswahl möglich)
- ☐ Anregung bzw. Initiierung
- ☐ Planung
- ☐ Gemeinsame Durchführung

5.3 Wenn ja, wie lautet der Name der studentischen Initiative(n)? (Mehrfachauswahl möglich)
- ☐ Health For Future
- ☐ PAN University
- ☐ UAEM
- ☐ IPPNW
- ☐ bvmd
- ☐ Sonstige

5.4 Wenn Sonstige, bitte Name(n) der studentischen Initiative(n) angeben:

☐

5.5 Gibt es im Rahmen der Veranstaltung Kooperationen mit Partnern innerhalb der medizinischen Fakultät?
- ☐ Ja
- ☐ Nein

5. Kooperationen Veranstaltung 1 [Fortsetzung]

5.6 Wenn ja, nennen Sie bitte den Kooperationspartner und beschreiben Sie die Art der Kooperation.

5.7 Gibt es im Rahmen der Veranstaltung Kooperationen mit Partnern außerhalb der medizinischen Fakultät?

☐ Ja ☐ Nein

5.8 Wenn ja, nennen Sie bitte den Kooperationspartner und beschreiben Sie die Art der Kooperation.

6. Lernziele Veranstaltung 1

*Die hier behandelten Themenbereiche nach unserer Definition von Lehre zu Planetarer Gesundheit basieren auf den Lernzielen des Themenkatalogs "Planetare und Globale Gesundheit" aus dem **NKLM**.*

6.1 Für welche der nebenstehenden Teilbereiche wird das folgende Lernziel erreicht?

Die Absolvent/innen verfügen über **Grundlegendes Wissen** über **anthropogene Umweltveränderungen.** (Mehrfachauswahl möglich)

- ☐ **Klimawandel**
- ☐ Weitere **Umweltveränderungen** (z.B. Primärwälder, Mikroplastik, Übernutzung Fischbestände)
- ☐ **Planetare Belastungsgrenzen** (Klimawandel, Einbringung neuartiger Substanzen und Organismen (inkl. Chemie- und Plastikverschmutzung), Ozonloch, Luftverschmutzung, Ozeanversauerung, Biogeochemische Kreisläufe, Süßwasserverbrauch, Abholzung und weitere Landnutzungsänderungen, Biodiversitätsverlust)
- ☐ **Systemforschung** (z.B. Kipppunkte, Nicht-Linearität, Rückkopplungsschleifen, Dringlichkeit zum Handeln)
- ☐ Keine
- ☐ Unbekannt

6.2 Für welche der nebenstehenden Teilbereiche wird das folgende Lernziel erreicht?

Die Absolvent/innen können die **Wechselwirkungen** zwischen Klima- und anderen anthropogenen **Umweltveränderungen** und **Gesundheit** erläutern. (Mehrfachauswahl möglich)

- ☐ **Hitze**/Hitzewellen
- ☐ Andere **Extremwetterereignisse**
- ☐ **Infektion**skrankheiten
- ☐ **Toxin**vermittelte Erkrankungen
- ☐ **Kardiovaskuläre** Erkrankungen
- ☐ **Endokrinologische** Erkrankungen
- ☐ **Onkologische** Erkrankungen
- ☐ **Allergien**
- ☐ **Mütter**- und **Kinder**gesundheit
- ☐ **Neurologische** Erkrankungen
- ☐ **Mentale** Gesundheit
- ☐ **Migration** und gewaltsame **Konflikte**
- ☐ Wechselwirkungen zwischen **Ernährung**, Gesundheit und Umwelt
- ☐ **Co-Benefits** (Gemeinsame/ Wechselseitige positive Effekte von Klima- und Gesundheitsschutz)
- ☐ Keine
- ☐ Unbekannt

6. Lernziele Veranstaltung 1 [Fortsetzung]

6.3 Werden Lernziele aus dem folgenden Bereich in Ihrer Veranstaltung verfolgt? ☐ Ja ☐ Nein ☐ Keine Angabe

Die Absolvent/innen verfügen über grundlegendes Wissen über **Bevölkerungsgruppen**, die in besonderer Weise von den Auswirkungen globaler **Umweltveränderungen betroffen** sind, und deren **Vulnerabilitätsfaktoren.**

Anmerkung zu 6.3:
*Dies umfasst **zum Beispiel:** Kinder, Säuglinge, alte Menschen, Pflegebedürftige, Schwangere, Frauen, Personen mit Vorerkrankungen, Multi-Medikation oder vorbestehender psychischer Vulnerabilität, Personen mit niedrigem sozioökonomischen Status oder ungünstigem Wohn-/Arbeits-/Lebensumfeld, Personen mit Migrationshintergrund, Bevölkerungen in Regionen ohne ausreichende soziale Sicherungssysteme, Prinzip der "Climate Justice" mit globalen und lokalen Diskrepanzen zwischen Verursachenden und Betroffenen*

6.4 Werden Lernziele aus dem folgenden Bereich in Ihrer Lehrveranstaltung verfolgt? ☐ Ja ☐ Nein ☐ Keine Angabe

Die Absolvent/innen reflektieren ihre **Verantwortung**, die menschliche **Gesundheit** und die dafür notwendigen natürlichen und gesellschaftlichen **Systeme** herzustellen, zu erhalten und zu **fördern**.

Anmerkung zu 6.4:
*Dies umfasst den Bereich "**Notwendige nachhaltige Transformation aller relevanten Gesellschaftsbereiche**" wie Mobilität, Ernährung, Energie, Agrar, Konsum, Wirtschaft, Gesundheitsversorgung sowie gesellschaftliche und rechtliche Werte und Normen.*

6.5 Welche Lernziele aus dem Bereich "Fertigkeiten zur **Umsetzung von Transformationsprozessen**" werden in Ihrer Veranstaltung verfolgt? (Mehrfachauswahl möglich)

☐ Klimakommunikation ☐ Wissenschaftskommunikation ☐ Transdisziplinäre Zusammenarbeit
☐ Projektmanagement ☐ Nachhaltigkeit im Gesundheitswesen ☐ Arbeit mit Hitzeaktionsplänen
☐ Keine ☐ Unbekannt

7. Lehrmethoden Veranstaltung 1

Bitte geben Sie den jeweiligen Anteil der Lehrmethode an der Lehrveranstaltung (nur Planetare Gesundheit) an.

		0% (nicht)	unter 50% (teilweise)	über 50% (überwiegend)	keine Angabe
7.1	Vortrag	☐	☐	☐	☐
7.2	Simulation/Planspiel	☐	☐	☐	☐
7.3	Training kommunikativer Kompetenzen: Gespräche mit Patient/innen oder Simulationspersonen	☐	☐	☐	☐
7.4	Training kommunikativer Kompetenzen: Gespräche mit Entscheidungstragenden	☐	☐	☐	☐

7. Lehrmethoden Veranstaltung 1 [Fortsetzung]

7.5	Training kommunikativer Kompetenzen: Gespräche mit Gesellschaft/persönlichem Umfeld	☐	☐	☐	☐
7.6	Kleingruppenarbeit mit Arbeitsauftrag	☐	☐	☐	☐
7.7	Selbststudium	☐	☐	☐	☐
7.8	Referate der Teilnehmenden	☐	☐	☐	☐
7.9	POL-Fälle (POL = Problemorientiertes Lernen)	☐	☐	☐	☐
7.10	Nutzen von vorbereiteten Unterrichtsmaterialien (Inverted Classroom)	☐	☐	☐	☐
7.11	Peer-Teaching	☐	☐	☐	☐

7.12 Weitere Lehrmethoden: Bitte Nennung inklusive Prozentzahl

8. Planetare Gesundheit und Klimawandel an Ihrer Fakultät

		Ja	Nein	keine Angabe
8.1	Gibt es an Ihrer Fakultät ein Institut, eine Abteilung oder eine Arbeitsgruppe für **Forschung** im Bereich Planetare Gesundheit?	☐	☐	☐

8.2 Wenn ja, was genau?

		Ja	Nein	keine Angabe
8.3	Gibt es an Ihrer Fakultät eine oder mehrere **spezifische beauftragte Person(en) für Lehre** im Bereich Planetare Gesundheit?	☐	☐	☐
8.4	Gibt es von Seiten der Fakultät spezifische **Förderprogramme für Lehre** im Bereich Planetare Gesundheit?	☐	☐	☐

8.5 Wenn ja, können Sie uns mehr darüber erzählen?

9. Weitere Lehrveranstaltungen

9.1 Möchten Sie im Folgenden die Fragen für eine zweite Lehrveranstaltung beantworten? ☐ Ja ☐ Nein

10. Grunddaten Veranstaltung 2

10. Grunddaten Veranstaltung 2 [Fortsetzung]

10.1 Titel der Veranstaltung

10.2 Wie viele Unterrichtseinheiten umfasst die Veranstaltung im gesamten Semester? (1 UE = 45 min)

☐ 1	☐ 2	☐ 3
☐ 4	☐ 5	☐ 6
☐ 7	☐ 8	☐ 9
☐ 10	☐ 11	☐ 12
☐ 13	☐ 14	☐ 15
☐ 16	☐ 17	☐ 18
☐ 19	☐ 20	☐ 21
☐ 22	☐ 23	☐ 24
☐ 25	☐ 26	☐ 27
☐ 28	☐ 29	☐ 30
☐ 31	☐ 32	☐ 33
☐ 34	☐ 35	☐ 36
☐ 37	☐ 38	☐ 39
☐ 40	☐ 41	☐ 42
☐ 43	☐ 44	☐ 45
☐ 46	☐ 47	☐ 48
☐ 49	☐ 50	☐ 51
☐ 52	☐ 53	☐ 54
☐ 55	☐ 56	☐ 57
☐ 58	☐ 59	☐ 60
☐ > 60	☐ Keine Angabe	

10.3 Seit wann besteht die Lehrveranstaltung in ihrer aktuellen Form?

☐ vor WS17/18	☐ WS17/18	☐ SS18
☐ WS18/19	☐ SS19	☐ WS19/20
☐ SS20	☐ WS20/21	☐ SS21
☐ WS21/22	☐ SS22	☐ WS22/23
☐ SS23 (geplant)	☐ Unbekannt	

10.4 Gab es in der Vergangenheit eine Vorgängerveranstaltung mit anderem Aufbau oder anderen Inhalten? ☐ Ja ☐ Nein

10.5 Wenn ja, seit wann bestand diese Vorgängerveranstaltung?

☐ vor WS17/18	☐ WS17/18	☐ SS18
☐ WS18/19	☐ SS19	☐ WS19/20
☐ SS20	☐ WS20/21	☐ SS21
☐ WS21/22	☐ SS22	☐ Unbekannt

*Bitte beachten Sie: Die nachfolgenden Fragen beziehen sich ausschließlich auf die **Veranstaltung in ihrer neuesten Form**.*

10.6 Welches Format hat die Lehrveranstaltung? ☐ Gesamter Kurs ("Course") ☐ Einzelveranstal-tung(en) innerhalb eines Kurses ("Class")

10.7 Falls es sich um (eine) einzelne Veranstaltung(en) innerhalb eines Kurses handelt, wie groß ist in etwa der Anteil am gesamten Kurs in Prozent?

☐ 0-20%	☐ 21-40%	☐ 41-60%
☐ 61-80%	☐ 81-100%	☐ keine Angabe

10. Grunddaten Veranstaltung 2 [Fortsetzung]

10.8 Wo ist die Veranstaltung im Curriculum verortet? (Mehrfachauswahl möglich)

- ☐ Curricular, Vorklinik
- ☐ Curricular, Klinik
- ☐ Wahlpflichtfach, Vorklinik
- ☐ Wahlpflichtfach, Klinik
- ☐ Extracurricular
- ☐ Bestandteil des Fachs "Umweltmedizin"
- ☐ Veranstaltung offen für Studierende weiterer medizinischer Fächer an der medizinischen Fakultät (z.B.: Zahnmedizin, Biomedizin, Tiermedizin)
- ☐ Veranstaltung offen für Studierende anderer Fakultäten

10.9 Wie hoch ist die Teilnehmendenzahl der Lehrveranstaltung im aktuellen Semester? (bzw. geplante Zahl, falls Beginn erst SS23)

- ☐ ≤ 10
- ☐ 11-20
- ☐ 21-30
- ☐ 31-40
- ☐ 41-50
- ☐ 51-60
- ☐ 61-70
- ☐ 71-80
- ☐ 81-90
- ☐ 91-100
- ☐ > 101
- ☐ Keine Angabe

10.10 Hier ist Platz für Ihre Anmerkungen zur Teilnehmendenzahl (z.B. Entwicklungen, Schwankungen, Unsicherheiten)

10.11 Welche Prüfungsmethode(n) kommt/kommen zur Anwendung? (Mehrfachauswahl möglich)

- ☐ Klausur (Multiple Choice)
- ☐ Klausur (Freitext)
- ☐ Mündliche Prüfung
- ☐ OSCE (Objective Structured Clinical Examination)
- ☐ Projektarbeit
- ☐ Abschlussreflexion/Präsentation
- ☐ Kurzaufsatz (Short Essay)
- ☐ Regelmäßige Teilnahme
- ☐ Unbekannt
- ☐ Sonstiges

10.12 Wenn Sonstiges, bitte Prüfungsmethode(n) ergänzen:

11. Kooperationen Veranstaltung 2

11.1 Ist an dem Lehrangebot eine studentische Initiative beteiligt?

- ☐ Ja
- ☐ Nein
- ☐ Unbekannt

11.2 Wenn ja, auf welche Weise? (Mehrfachauswahl möglich)

- ☐ Anregung bzw. Initiierung
- ☐ Planung
- ☐ Gemeinsame Durchführung

11.3 Wenn ja, wie lautet der Name der studentischen Initiative(n)? (Mehrfachauswahl möglich)

- ☐ Health For Future
- ☐ PAN University
- ☐ UAEM
- ☐ IPPNW
- ☐ bvmd
- ☐ Sonstige

11.4 Wenn Sonstige, bitte Name(n) der studentischen Initiative(n) angeben:

11.5 Gibt es im Rahmen der Veranstaltung Kooperationen mit Partnern innerhalb der medizinischen Fakultät?

- ☐ Ja
- ☐ Nein

11.6 Wenn ja, nennen Sie bitten den Kooperationspartner und beschreiben Sie die Art der Kooperation.

11. Kooperationen Veranstaltung 2 [Fortsetzung]

11.7 Gibt es im Rahmen der Veranstaltung Kooperationen mit Partnern außerhalb der medizinischen Fakultät?

☐ Ja ☐ Nein

11.8 Wenn ja, nennen Sie bitten den Kooperationspartner und beschreiben Sie die Art der Kooperation.

12. Lernziele Veranstaltung 2

*Die hier behandelten Themenbereiche nach unserer **Definition von Lehre zu Planetarer Gesundheit** basieren auf den Lernzielen des Themenkatalogs "Planetare und Globale Gesundheit" aus dem **NKLM**.*

12.1 Für welche der nebenstehenden Teilbereiche wird das folgende Lernziel erreicht?

Die Absolvent/innen verfügen über **Grundlegendes Wissen** über **anthropogene Umweltveränderungen.** (Mehrfachauswahl möglich)

- ☐ **Klimawandel**
- ☐ Weitere **Umwelteinflüsse** (z. B. Primärwälder, Mikroplastik, Übernutzung Fischbestände)
- ☐ **Planetare Belastungsgrenzen** (Klimawandel, Einbringung neuartiger Substanzen und Organismen (inkl. Chemie- und Plastikverschmutzung), Ozonloch, Luftverschmutzung, Ozeanversauerung, Biogeochemische Kreisläufe, Süßwasserverbrauch, Abholzung und weitere Landnutzungsänderungen, Biodiversitätsverlust)
- ☐ **Systemforschung** (z.B. Kipppunkte, Nicht-Linearität, Rückkopplungsschleifen, Dringlichkeit zum Handeln)
- ☐ Keine
- ☐ Unbekannt

12.2 Für welche der nebenstehenden Teilbereiche wird das folgende Lernziel erreicht?

Die Absolvent/innen können die **Wechselwirkungen** zwischen Klima- und anderen anthropogenen **Umweltveränderungen** und **Gesundheit** erläutern. (Mehrfachauswahl möglich)

- ☐ **Hitze**/Hitzewellen
- ☐ Andere **Extremwetterereignisse**
- ☐ **Infektion**skrankheiten
- ☐ **Toxin**vermittelte Erkrankungen
- ☐ **Kardiovaskuläre** Erkrankungen
- ☐ **Endokrinologische** Erkrankungen
- ☐ **Onkologische** Erkrankungen
- ☐ **Allergien**
- ☐ **Mütter**- und **Kinder**gesundheit
- ☐ **Neurologische** Erkrankungen
- ☐ **Mentale** Gesundheit
- ☐ **Migration** und gewaltsame **Konflikte**
- ☐ Wechselwirkungen zwischen **Ernährung**, Gesundheit und Umwelt
- ☐ **Co-Benefits** (Gemeinsame/ Wechselseitige positive Effekte von Klima- und Gesundheitsschutz)
- ☐ Keine
- ☐ Unbekannt

12. Lernziele Veranstaltung 2 [Fortsetzung]

12.3 Werden Lernziele aus dem folgenden Bereich in Ihrer Veranstaltung verfolgt? ☐ Ja ☐ Nein ☐ Keine Angabe

Die Absolvent/innen verfügen über grundlegendes Wissen über **Bevölkerungsgruppen**, die in besonderer Weise von den Auswirkungen globaler **Umweltveränderungen betroffen** sind, und deren **Vulnerabilitätsfaktoren.**

Anmerkung zu 12.3:
*Dies umfasst **zum Beispiel:** Kinder, Säuglinge, alte Menschen, Pflegebedürftige, Schwangere, Frauen, Personen mit Vorerkrankungen, Multi-Medikation oder vorbestehender psychischer Vulnerabilität, Personen mit niedrigem sozioökonomischen Status oder ungünstigem Wohn-/Arbeits-/Lebensumfeld, Personen mit Migrationshintergrund, Bevölkerungen in Regionen ohne ausreichende soziale Sicherungssysteme, Prinzip der "Climate Justice" mit globalen und lokalen Diskrepanzen zwischen Verursachenden und Betroffenen*

12.4 Werden Lernziele aus dem folgenden Bereich in Ihrer Lehrveranstaltung verfolgt? ☐ Ja ☐ Nein ☐ Keine Angabe

Die Absolvent/innen reflektieren ihre **Verantwortung**, die menschliche **Gesundheit** und die dafür notwendigen natürlichen und gesellschaftlichen **Systeme** herzustellen, zu erhalten und zu **fördern**.

Anmerkung zu 12.4:
*Dies umfasst den Bereich "**Notwendige nachhaltige Transformation aller relevanten Gesellschaftsbereiche**" wie Mobilität, Ernährung, Energie, Agrar, Konsum, Wirtschaft, Gesundheitsversorgung sowie gesellschaftliche und rechtliche Werte und Normen.*

12.5 Welche Lernziele aus dem Bereich "Fertigkeiten zur **Umsetzung von Transformationsprozessen**" werden in Ihrer Veranstaltung verfolgt? (Mehrfachauswahl möglich)

- ☐ Klimakommunikation
- ☐ Wissenschaftskommunikation
- ☐ Transdisziplinäre Zusammenarbeit
- ☐ Projektmanagement
- ☐ Nachhaltigkeit im Gesundheitswesen
- ☐ Arbeit mit Hitzeaktionsplänen
- ☐ Keine
- ☐ Unbekannt

13. Lehrmethoden Veranstaltung 2

Bitte geben Sie den jeweiligen Anteil der Lehrmethode an der Lehrveranstaltung (nur Planetare Gesundheit) an.

	0% (nicht)	unter 50% (teilweise)	über 50% (überwiegend)	keine Angabe
13.1 Vortrag	☐	☐	☐	☐
13.2 Simulation/Planspiel	☐	☐	☐	☐

13. Lehrmethoden Veranstaltung 2 [Fortsetzung]

13.3 Training kommunikativer Kompetenzen: Gespräche mit Patient/innen oder Simulationspersonen	☐	☐	☐	☐
13.4 Training kommunikativer Kompetenzen: Gespräche mit Entscheidungstragenden	☐	☐	☐	☐
13.5 Training kommunikativer Kompetenzen: Gespräche mit Gesellschaft/persönlichem Umfeld	☐	☐	☐	☐
13.6 Kleingruppenarbeit mit Arbeitsauftrag	☐	☐	☐	☐
13.7 Selbststudium	☐	☐	☐	☐
13.8 Referate der Teilnehmenden	☐	☐	☐	☐
13.9 POL-Fälle (POL = Problemorientiertes Lernen)	☐	☐	☐	☐
13.10 Nutzen von vorbereiteten Unterrichtsmaterialien (Inverted Classroom)	☐	☐	☐	☐
13.11 Peer-Teaching	☐	☐	☐	☐

13.12 Weitere Lehrmethoden: Bitte Nennung inklusive Prozentzahl

14. Abschluss

Herzlichen Dank für Ihre Angaben!

Nachdem Sie die Umfrage durch **Klicken auf das Feld "Absenden"** beendet haben, werden Sie zu einer zweiten Umfrage weitergeleitet. Wir würden uns freuen, wenn Sie dort Ihre Kontaktdaten eintragen, falls Sie an weiterer Vernetzung interessiert sind.

Die Sicherung Ihrer Daten war erst erfolgreich, wenn Sie zur zweiten Umfrage weitergeleitet wurden.

Wenn Sie von weiteren Lehrveranstaltungen zur Planetaren Gesundheit an Ihrer Fakultät wissen, **teilen Sie gerne diese Umfrage** mit Ihren Kolleginnen und Kollegen.
Falls Sie die Umfrage selbst für weitere Lehrveranstaltungen ausfüllen möchten, starten Sie sie bitte unter dem Ihnen zugesendeten Link erneut.

14.1 Möchten Sie uns noch etwas mitteilen?

English translation of the questionnaire

TEMPLATE

Please note: This is a translated and shortened version of the online-questionnaire submitted to the participants during the PlanetMedEd-Study. Further explanations are indicated via square brackets [].

EvaSys	Planetary Health Education	Electric Paper

1. Introduction

Dear participant,

The working group "Climate and Planetary Health" of the Faculty of Medicine in Würzburg would like to assess teaching and learning activities in the field of **planetary health (climate, environment, health)** with your help as part of the PlanetMedEd study. The current status will help to provide an overview and thus provide important orientation in the (further) development of courses.

Answering the questions will take approx. **15 minutes**. If there are **no corresponding offers** at your faculty, please also let us know briefly in the questionnaire. In this case, answering the questions will take approx. **2 minutes**.

You are welcome to describe various courses in this survey. Please answer the questions **first for one of the courses only**. Afterwards, you will be able to answer the questions for a second course. This will require an additional 10 minutes.

If you have any questions, please contact Dr. med. Eva-Maria Schwienhorst-Stich (Schwienhor_E@ukw.de) or the doctoral student Fabio Grieco (fabio.grieco@stud-mail.uni-wuerzburg.de).

We would like to thank you very much in advance for your cooperation!
For a smooth process, pop-up blockers should be deactivated in your browser.

2. Data protection information

Below you will find information on data protection in this study. Your consent is a prerequisite for participation in the study.
The data of the survey is collected **anonymously**. By participating, you agree to the storage and evaluation of the data for research purposes as well as to the possible publication of the data. Your **participation** in the study is **voluntary** and can be discontinued at any time. By participating, you agree to the data protection information.

[In the following, participants found detailed information on data protection according to European law.]

2.1 I have read the above information and have read the instructions. I have had enough time to decide to participate and agreed to participate. ☐ Yes

3. General information

3.1 For which medical school (location) are you answering this questionnaire?

[Participants could select their medical school from a list]

3.2 In which organisational unit or in which function do you work at the Faculty of Medicine? ☐ Deanery Study deanery ☐ Lecturer ☐ Other

3.3 If you are a lecturer, at which clinic/institute/for which subject?

TEMPLATE

3.4 If other, please specify (e.g. clinic/institute, exact function)

3.5 Does your faculty offer any courses on planetary health up to winter semester 22/23 or is there a course planned for summer semester 23? ☐ Yes ☐ No

Here you will find a definition of planetary health education. Courses can be included in the survey if at least one aspect from 1.1 AND 1.2 is taught.

[By clicking on a link, participants could access the *Definition of Planetary Health topics* used within the PlanetMedEd-Study]

3.6 Is there an institute, a department or a working group for research in the field of planetary health at your medical school? ☐ Yes ☐ No ☐ No information

3.7 If yes, please specify

4. Information on course 1

4.1 Course title:

Please fill in the questionnaire right now for one course only. Afterwards, you will have the opportunity to fill in the questionnaire for a second course.

4.2 How many course units does the course comprise (one CU = 45 minutes)?

[Participants could select a number between one and 60, the option > 60 and "no information".]

4.5 When was the course firstly introduced in its current format?

- ☐ prior to WS17/18
- ☐ WS17/18
- ☐ SS18
- ☐ WS18/19
- ☐ SS19
- ☐ WS19/20
- ☐ SS20
- ☐ WS20/21
- ☐ SS21
- ☐ WS21/22
- ☐ SS22
- ☐ WS22/23
- ☐ SS23 (planned)
- ☐ Unknown

4.4 ☐ Yes ☐ No

TEMPLATE

Was there a preceding course in the past with different structure or content?

4.5 If yes, when was this course first introduced?

☐ prior to WS17/18	☐ WS17/18	☐ SS18
☐ WS18/19	☐ SS19	☐ WS19/20
☐ SS20	☐ WS20/21	☐ SS21
☐ WS21/22	☐ SS22	☐ Unknown

Please note: The following questions refer exclusively to the **course in its latest form**.

4.6 What is the format of the course?

☐ Stand-alone course („course")
☐ Individual sessions within a course ("Class")

4.7 If it is a single session within a course, how large is the share of planetary health content in the whole course?

☐ 0-20%	☐ 21-40%	☐ 41-60%
☐ 61-80%	☐ 81-100%	☐ no answer

4.8 Where in the curriculum is your course located? (Multiple selection possible)

[Participants could select from the following options: curricular, environmental medicine, elective (clinical study part), elective (preclinical study part), extracurricular, open to other students of the medical faculty (e.g. dentistry, biomedicine, veterinary medicine), open for students of other faculties]

TEMPLATE

4.9 What is the number of course participants in the current semester? (or planned, if the start is summer 23)

- ☐ ≤ 10
- ☐ 11-20
- ☐ 21-30
- ☐ 31-40
- ☐ 41-50
- ☐ 51-60
- ☐ 61-70
- ☐ 71-80
- ☐ 81-90
- ☐ 91-100
- ☐ > 101
- ☐ No information

4.10 Here you can comment on the number of participants (e.g. developments, uncertainties).

4.11 Which examination method(s) are used? (Multiple selection possible)

- ☐ MC-exam
- ☐ Open-text-exam
- ☐ Oral exam
- ☐ OSCE (Objective Structured Clinical Examination)
- ☐ Project work
- ☐ Final reflection/presentation
- ☐ Short Essay
- ☐ Regular Attendance
- ☐ Unknown
- ☐ Other

4.12 If other, please specify:

5. Collaboration

5.1 Is there any collaboration with student initiatives? ☐ Yes ☐ No ☐ Unknown

5.2 If yes, how? (Multiple selection possible)

- ☐ Initiation
- ☐ Planning
- ☐ Implementation

5.3 If yes, what is/are the name(s) of the student initiative(s)? (Multiple selection possible)

- ☐ Health For Future
- ☐ PAN University
- ☐ UAEM
- ☐ IPPNW
- ☐ bvmd
- ☐ Other

5.4 If other, please specify:

5.5 Is there any cooperation with partners within the medical school? ☐ Yes ☐ No

5.6 If yes, please name the partner and describe the type of cooperation.

5.7 Is there any cooperation with partners outside the medical school? ☐ Yes ☐ No

TEMPLATE

5.8 If yes, please name the partner and describe the type of cooperation.

6. Learning Objectives

The topics covered here in line with our definition of teaching on planetary health are based on the learning objectives of the addendum on "Planetary and Global Health" of the **NKLM**

[German National Competency-based Learning Objectives Catalogue in Medicine, by clicking on a link, participants could access the *Definition of Planetary Health topics* used within the PlanetMedEd-study. For clarity, this part of the questionnaire is shortened, all items are displayed in table 2].

6.1 Which of the following learning objectives are addressed in the course?

[participants could select learning objectives from chapter 1.1: climate change, other environmental changes, planetary boundaries, systems research as well as the options "none" or "unknown"]

6.2 Which of the following learning objectives are addressed in the course?

[participants could select learning objectives from chapter 1.2: heat, other extreme weather events, infectious diseases, toxin-mediated diseases, cardiovascular diseases, endocrinological diseases, oncological diseases, allergies, maternal and child health, neurological diseases, mental health, migration and violent conflicts, connections of nutrition, environment and health, co-benefits as well as the options "none" or "unknown"]

6.3 Are there any learning objectives from this list addressed in your course?

[participants were shown the learning objectives from chapter 1.3: populations who are especially affected by global environmental changes and their vulnerability factors They could select the options "yes", "no" and "no information".]

6.4 Are there any learning objectives from this list addressed in your course?

[participants were shown the learning objectives from chapter 2: responsibility to establish, maintain and foster human health and the natural and social systems on which it depends
They could select the options "yes", "no" and "no information".]

6.5 Which of the following learning objectives are achieved in your course?

[participants could select learning objectives from chapter 3: climate communication, science communication, transdisciplinary collaboration, project management, sustainable healthcare, working with heat action plans as well as the options "none" or "unknown"]

TEMPLATE

7. Teaching Methods

[In this chapter, participants were given the following list of teaching methods: lecture, simulation, skills training for communication with patients, skills training for communication with decision makers, skills training for communication with society, small group work, self-study, presentations, PBL-cases, inverted classroom, peer teaching.

For each method, they should select whether it was used predominantly (> 50%), partly (<50%) or not at all (0%) during the course. They could also select "no information". They could also submit other teaching methods including the percentage of those other methods.]

8. Institutional Support

8.1 Is there a research working group focusing on planetary health at your institution ☐ Yes ☐ No, ☐ No information

8.2 If yes, please specify

8.3 Is there a dedicated coordinator for planetary health education at your institution? ☐ Yes ☐ No, ☐ No information

8.4 Is there a dedicated funding programme for planetary health education at your institution? ☐ Yes ☐ No, ☐ No information

8.5 If yes, please provide more information

Would you like to fill in the survey for a second course? ☐ Yes ☐ No

[If participants answered Yes, sections 3 to 7 could be filled in for a second course].

Conclusion

Thank you very much for providing your valuable information! After you finish the survey by **clicking "submit"**, you will be redirected to a second survey. We would be pleased if you entered your contact details there if you are interested in further networking.

Saving your survey data was only successful after you have been redirected to the second survey.

If you know of any other courses on planetary health at your faculty, **please feel free to share this survey** with your colleagues. If you would like to complete the survey yourself for further courses, please start it again using the link sent to you.

5 Sampling Strategy (Online Survey)

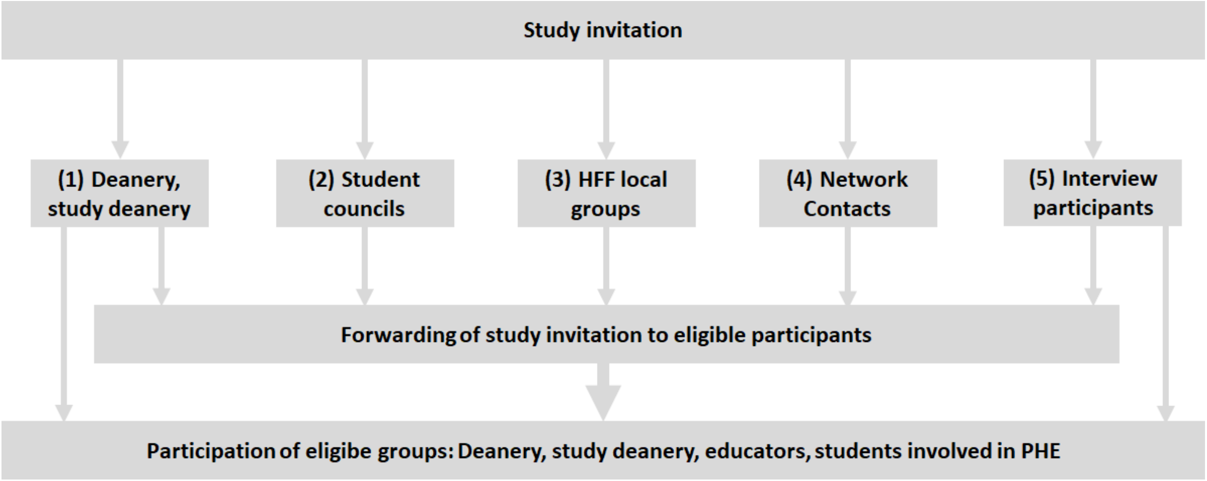

Supplementary Figure 2. Sampling strategy (online survey). Nationwide network contacts (4) included: German Alliance on Climate Change and Health (KLUG e.V.), Health For Future (HFF) and Master of Medical Education (MME) networks
